# Fertility in a high-altitude environment is compromised by luteal dysfunction: the relative roles of hypoxia and oxidative stress

**DOI:** 10.1186/1477-7827-11-24

**Published:** 2013-03-23

**Authors:** Víctor H Parraguez, Bessie Urquieta, Laura Pérez, Giorgio Castellaro, Mónica De los Reyes, Laura Torres-Rovira, Adriana Aguado-Martínez, Susana Astiz, Antonio González-Bulnes

**Affiliations:** 1 Faculty of Veterinary Sciences, University of Chile, Casilla 2, Correo 15, La Granja, Santiago, Chile; 2 Faculty of Agricultural Sciences, University of Chile, Casilla 2, Correo 15, La Granja, Santiago, Chile; 3 International Centre for Andean Studies, University of Chile, Casilla 2, Correo 15, La Granja, Santiago, Chile; 4 Department of Animal Biology, University of Sassari, Via Vienna 2, Sassari, 07100, Italy; 5 SALUVET, Animal Health Department, Faculty of Veterinary Sciences, Complutense University of Madrid, Ciudad Universitaria s/n, Madrid, 28040, Spain; 6 Department of Animal Reproduction, INIA, Av. Puerta de Hierro s/n, Madrid, 28040, Spain

**Keywords:** Sheep, Corpus luteum function, Hypoxia, Oxidative stress, Antioxidant vitamins

## Abstract

**Background:**

At high altitudes, hypoxia, oxidative stress or both compromise sheep fertility. In the present work, we tested the relative effect of short- or long-term exposure to high altitude hypobaric hypoxia and oxidative stress on corpora luteal structure and function.

**Methods:**

The growth dynamics of the corpora lutea during the estrous cycle were studied daily by ultrasonography in cycling sheep that were either native or naïve to high-altitude conditions and that were supplemented or not supplemented with antioxidant vitamins. Arterial and venous blood samples were simultaneously drawn for determination of gases and oxidative stress biomarkers and progesterone measurement. On day five after ovulation in the next cycle, the ovaries were removed for immunodetection of luteal HIF-1alpha and VEGF and IGF-I and to detect IGF-II gene expression.

**Results:**

The results showed that both short- and long-term exposure to high-altitude conditions decreased luteal growth and IGF-I and IGF-II gene expression but increased HIF-1 alpha and VEGF immunoexpression. The level of plasma progesterone was also increased at a high altitude, although an association with increased corpus luteum vascularization was only found in sheep native to a high-altitude location. Administration of antioxidant vitamins resulted in a limited effect, which was restricted to decreased expression of oxidative stress biomarkers and luteal HIF-1alpha and VEGF immunoexpression.

**Conclusions:**

Exposure of the sheep to high-altitude hypobaric hypoxia for short or long time periods affects the development and function of the corpus luteum. Moreover, the observed association of oxidative stress with hypoxia and the absence of any significant effect of antioxidant vitamins on most anatomical and functional corpus luteum traits suggests that the effects of high altitude on this ovarian structure are mainly mediated by hypoxia. Thus, these findings may help explain the decrease in sheep fertility at a high altitude.

## Background

In humans [1] and domestic animal species living in high-altitude environments, such as sheep introduced to high-plateaus [2,3], the fertility of females is reduced when compared to their low-altitude counterparts.

The sociological impact of this phenomenon has encouraged the study of its causal factors because approximately 140 million people live at altitudes higher than 2500 meters above sea level (m.a.s.l.). However, most of the data obtained have been acquired by observational studies, which are usually biased by concurrent factors related to economic impoverishment and malnutrition, as well as behavioral and socio-cultural factors specific to human populations [4]. Thus, research methods must isolate the physiological causes from these concurrent factors. Such an objective can only be accomplished by interventional studies under well-determined conditions, making the use of animal models indispensable.

In this scenario, sheep have a prominent role with a dual purpose. First, from a production perspective, sheep are a major economical resource for approximately 25 million rural people living above 2500 m.a.s.l. in developing regions and transition countries, such as the Andean and Qinghai-Tibetan high-plateaus [5]. Second, sheep provide a widely recognized animal model for biomedical studies.

European settlers introduced sheep to the Andean highlands approximately 500 years ago. Despite this lengthy period of time for adaptation to high-altitude conditions, the reproductive efficiency remains poor, with a lamb:ewe proportion of approximately 0.4 in the Chilean Northern plateau [3]. Such low reproductive efficiency is even more marked in ovine newcomers to a high altitude [2], which limits the application of genetic improvement programs to introduce selected animals. The same effect after acute exposure to hypoxia has been described in human beings, but comparative studies of high- and low-altitude populations are controversial because of difficulties in separating the associated behavioral and sociocultural factors [4]. This situation reinforces the necessity of research in this area.

A number of different factors may affect female fertility by affecting the functionality of the hypothalamus-hypophysis-ovarian axis, cyclic ovulatory activity, the quality of preovulatory follicles/oocytes/embryos and/or subsequent embryo/fetal viability. All of these critical roles require the presence of a fully functional corpus luteum.

In recent experiments, we demonstrated that the deleterious effects of hypoxia-induced oxidative stress at a high altitude may be prevented by daily administration of antioxidant vitamins, which increase the plasma progesterone concentrations throughout pregnancy [6] and improve placental structure and fetal growth [7]. In addition, empirical observations have shown that antioxidant supplementation increases fertility in cycling ewes at high altitudes [2]. These findings prompted us to hypothesize that low fertility in ewes at a high altitude may result from inadequate corpus luteum function, which may be caused by hypoxia, oxidative stress or both. Thus, the objective of the present experiment was to rigorously study the effects of high altitude (comparing ewes exposed to high altitude with those not exposed to a high altitude) and the altitudinal status (comparing ewes native to a high altitude region with those naïve to high altitude) on the pattern of growth, vascularization and secretory activity of the corpora lutea throughout the estrous cycle, as well as on the expression of key factors involved in the growth and vascularization of the corpora lutea (*HIF-1, VEGF, IGF-I* and *IGF-II*). At the same time, we aimed to determine the effects of antioxidant therapy with vitamins C and E on the structure and function of the corpora lutea at different altitudes and altitudinal statuses.

## Methods

### Animals and management

This study was performed in agreement with the International Guiding Principles for Biomedical Research Involving Animals (Council for International Organization of Medical Sciences, World Health Organization), and the study was approved by the Bioethics Review Committee of the Faculty of Veterinary and Animal Sciences, University of Chile, as well as by the Bioethics Advisory Committee of the Chilean National Commission for Scientific and Technological Research (CONICYT, Chile).

In total, 36 multiparous Creole ewes (a Chilean mixed breed developed from Churra and Manchega Spanish breeds) with a history of normal pregnancies and deliveries were used. Twelve of the sheep (group HH) were native to high altitudes (descendants of sheep introduced to the Andean high plateau by Spanish settlers almost 500 years ago). At the beginning of the experiment, these ewes were kept together in a single pen at the animal facilities of the International Centre for Andean Studies (INCAS, University of Chile; 3600 m.a.s.l., barometric pressure ~ 667 hPa). The other 24 ewes were native to a low altitude condition (500 m.a.s.l., barometric pressure ~ 990 hPa) and selected on the basis of similar phenotypes, body weights and age as the females in the HH group. Half of these animals (LH group, n=12) were moved to the INCAS facilities to join the HH ewes. The remaining 12 ewes were maintained at a low altitude (LL group). The animals were provided with alfalfa hay daily (2 kg/animal/day, DM=89.8%, ME=10.6 MJ/kg, CP=14.0%) and fresh water *ad libitum*. The food supply was calculated to satisfy the daily ovine requirements in late gestation [8].

To determine the effects of the antioxidant treatment, the ewes in each group (HH, LH and LL) were randomly divided into two equal subgroups that were allocated in two different pens. Half of the animals remained as controls (maintaining the identification HH, LL and LH; n= 6 each), whereas the other half of the sheep were treated daily with antioxidant vitamins (groups HHV, LHV and LLV; n= 6 each) with an individual ration of 0.3 kg of alfalfa supplemented with 500 mg of vitamin C and 350 I.U. of vitamin E early in the morning every day. After consumption of the alfalfa supplemented with vitamins, the ration was raised to 2 kg/animal/day, as in the control group.

In summary, six experimental groups were formed. Four of them were kept at high altitudes: group HH (n=6, ewes native to a high altitude, without vitamin supplementation), group HHV (n=6, ewes with the same altitudinal status as HH but supplemented with antioxidant vitamins), group LH (n=6, ewes native to sea level and taken to high altitudes, without vitamin supplementation) and group LHV (n=6, ewes with the same altitudinal status as LH, supplemented with vitamins). Two additional groups of ewes native to a low altitude were maintained at a low altitude; one of them received vitamin supplementation (group LLV, n=6), and the other did not (group LL, n=6).

### Experimental procedure

On the same day that the antioxidant treatment started, the sheep were anesthetized (20 mg/kg i.m. of ketamine clorhidrate; Ketamil®, Troy Laboratories, Smithfield, Australia) for the installation of two catheters (2.5 mm internal diameter, Tygon®, Saint Gobain Performance Plastics, Akron, Ohio, U.S.A.) into the left pedal artery and vein. Once installed, the catheters were filled with heparinized saline (1000 IU/mL) to prevent clot formation. The catheters were then passed through the subcutaneous tissue to the left flank of the ewe where they were exteriorized and placed in a canvas pocket attached to the skin.

On the day after initiation of the antioxidant treatment, the estrus cycles were synchronized by the administration of two i.m. doses of 125 μg of cloprostenol (Ovolute®, Drag Pharma, Santiago, Chile) given 9 days apart. Estrus detection was performed daily with vasectomized trained males. Two vasectomized rams with their chests painted with a mixture of vegetable oil and colored powder remained with the females in each one of the pens. On the following day, any ewe in heat was marked and identified.

The *in vivo* experimental sampling (for the study of oxygenation status, the incidence of oxidative stress, the pattern of growth and the activity of the corpora lutea) was performed during the second estrous cycle after the prostaglandin treatment. The *ex vivo* experimental sampling (for the study of gene or protein expression of different factors and vascular areas of the corpora lutea) was performed during the subsequent estrous cycle (i.e., the third cycle after prostaglandin treatment).

### Measurement of oxygenation status

These measurements were taken from arterial blood samples (1 mL) collected precisely at the beginning of the second estrous cycle after prostaglandin synchronization. Each day, the oxygenation status was determined using the partial pressure of oxygen (P_a_O_2_), the partial pressure of carbon dioxide (P_a_CO_2_), the hematocrit value (Ht), the hemoglobin concentration (Hb), the Hb saturation by oxygen (SatHb) and the pH value. These measurements were performed in an IL Synthesis 25™ gas analyzer (Instrumentation Laboratory, Lexington, MA, U.S.A.) calibrated to the local atmospheric pressure and ovine body temperature.

### Evaluation of oxidative stress

Oxidative stress was evaluated by measurement of oxidative damage biomarkers for proteins (carbonyl groups, CO) and lipids (malondialdehyde, MDA). The CO levels were determined by spectrophotometry, following the protocol described by Reznick and Packer [9]. MDA was measured by HPLC with fluorescence detection using the thiobarbituric acid assay described by Lastard *et al*. [10]. In addition, the total antioxidant capacity in the plasma (TAC) was assessed through the Total Radical-Trapping Antioxidant Parameter technique described by Wayner *et al*. [11] and modified by Lissi *et al*. [12].

### Determination of the growth pattern of the corpora lutea

The evaluations of the preovulatory follicle size and the growth of the corpus luteum were performed by ultrasonography using a real time B-mode ultrasound (Aloka SSD 500, Tokyo, Japan) fitted to a 7.5 MHz transrectal probe (Aloka UST 600–7.5, Tokyo, Japan). In brief, the observations were conducted with the sheep placed in dorsal recumbence on a metal cradle used for laparoscopy. Each ovary was scanned several times from different angles to obtain the images showing the largest cross-sectional area of each preovulatory follicle and the corpus luteum. Afterwards, preovulatory follicles and corpora lutea sizes were measured using the built-in electronic calipers and the area measurement tool of the ultrasound machine.

### Evaluation of luteal activity

The progesterone concentrations in the peripheral blood were determined daily in the venous blood plasma during the second estrous cycle after prostaglandin synchronization. Blood samples (5 mL) were obtained in heparinized syringes and centrifuged at 800 g × 5 min. The plasma was stored at −20°C until measurement. Progesterone was analyzed using a solid phase radioimmunoassay (RIA) specific for this steroid using reagents and techniques provided by DPC® Coat-a-Count® (Diagnostic Corporation, Los Angeles, CA, USA). The samples were measured in duplicate, and the assay sensitivity was 0.1 nM (0.031 ng/mL). The intra- and inter-assay coefficients of variation were 4.9 and 8.5%, respectively.

### Sampling for the evaluation of growth factor expression and the vascular area of the corpora lutea

At Day 5 of the third cycle after prostaglandin treatment, the ovary in which ovulation occurred was surgically removed laparoscopically under deep anesthesia. After removing each ovary, a small sample of luteal tissue was obtained and stored in liquid nitrogen for the study of IGF-I and IGF-II gene expression by quantitative reverse transcriptase PCR (qRT-PCR). The rest of the ovary was dissected into two parts through the center of the corpus luteum and immersed in a fixative solution (4% buffered paraformaldehyde) for 24 hours at room temperature. After a brief post-fixative period, the ovaries were processed for paraffin-embedded immunohistochemical study of HIF-1α and VEGF protein expression and for conventional hematoxylin–eosin (H–E) staining.

### IGF-I and IGF-II gene expression quantification

To determine the transcription levels of these key genes in the luteal tissues, the mRNA levels were analyzed by real-time qRT-PCR. The total RNA was extracted from 50 mg of tissue using Tri-ReagentTM (Sigma-Aldrich, Madrid, Spain), according to the manufacturer’s instructions. Traces of contaminating genomic DNA were removed from the RNA samples by treatment with rDNase I, and reaction rests removed using a DNA-*free*™ kit (Fischer Scientific-Life Technologies, Madrid, Spain).

The obtained RNA was quantified using a NanoDrop spectrophotometer (NanoDrop Technologies, Wilmington, USA), and the RNA quality was assessed by electrophoresis in an agarose gel. cDNA was synthesized from the total RNA using a SuperScript® VILO™ cDNA Synthesis Kit (Life Technologies, Madrid, Spain), according to the manufacturer’s instructions.

Specific forward and reverse primers were designed to amplify 60–200 bp target transcripts from IGF-I, IGF-II and β-actin (housekeeping) ovine genes available in Genbank, using the Primer3Plus program [13]; the sequences are shown in Table 1.

**Table 1:** Primers sequences used for IGF-I, IGF-II and β-actin ovine genes amplification

**Gen**	**Forward-primer**	**Reverse-primer**	**Accession Nº**	**Amplicon length**
*β-actin*	agggctcaggaatccatctt	ggaagctggtgcaggtagag	BC102948	79
*IGF-I*	accctccagtttgtctgtgg	agggtcatttttgcaaggtg	M30653	99
*IGF-II*	accctccagtttgtctgtgg	acacatccctctcggacttg	M89788	166

Standard PCR amplification was performed, and the amplified products were visualized on 8% agarose gels (Agarosa MS-8; Conda Pronadisa, Torrejón de Ardoz, Madrid, Spain). Real-time PCR for each target sequence was performed using 5 μL of cDNA diluted 1:100 using Power SYBR® Green PCR Master Mix (Applied Biosystems, Foster City, California, USA) and the corresponding forward and reverse primers in the ABI Prism 7000 Sequence Detector (Applied Biosystems). As negative controls, RNA samples were processed using the above procedure. The cycling conditions were 95°C for 10 min, followed by 40 cycles of 95°C (15 s) and 60°C (1 min). Single peaks in the dissociation curves confirmed the specific amplification of the selected genes. All samples were processed in duplicate, and the standard deviation (SD) of the threshold cycle (Ct) values for each gene did not exceed 0.5.

The results of each reaction were expressed as the relative transcription level of each gene after conversion of the raw mean Ct values to the linear form 2^-ΔΔCt^[14]. The ΔCt was calculated as the difference between the mean Ct value of each target gene transcript and the ovine β-actin transcript (housekeeping) for each sample.

### Immunohistochemical evaluation of HIF-1α and VEGF

Serial transverse sections of 7-μm thickness were obtained from each half-ovary. Three alternate sections were mounted on silanized slides for immunohistochemical procedures. After tissue rehydration, antigen unmasking was performed by microwaving in 0.1 M sodium citrate solution. The endogenous peroxidase activity was quenched with 3% hydrogen peroxide for 30 min, and the sections were gently washed in TBS (for HIF-1α) or PBS (for VEGF). Nonspecific binding was blocked by incubation with 10% non-immune goat serum in TBS (HIF-1α) or 1% non-immune bovine serum albumin (BSA) in PBS (VEGF) for 30 min.

The tissue sections were incubated for 12 h at 4°C with an antibody against HIF-1α (H1alpha67; Abcam, Cambridge, UK) diluted 1:100 in goat serum or against VEGF (sc-152; Santa Cruz Biotechnology, Santa Cruz, California, USA) diluted 1:200 in PBS and 1% BSA. After washing with the appropriate buffered solutions, the sections were incubated for 1 h at room temperature with a biotinylated goat antibody against mouse (ab6788, Abcam, Cambridge, UK) diluted 1:100 in goat serum or with a goat antibody against rabbit (sc-2040; Santa Cruz Biotechnology) diluted 1:200 in PBS for HIF-1α and VEGF, respectively. Then, a complex of streptavidin–peroxidase (SA202; Chemicon) was added. The reaction was visualized by adding 3,3-diaminobenzidine (DAB) to a working solution of a DAB Substrate Kit for Peroxidase (SK-4100; Vector Laboratories, Burlingame, California, USA) for 5 min. The sections were then washed, dehydrated and mounted using BIO-G medium (MCH30; Prolab, Santiago, Chile). Nonspecific background staining from the secondary antibody was tested by omission of the primary antibody from the control sections and replacement with BSA. The staining for each specific protein was evaluated by light microscopy (5 fields per section, randomly selected) at a magnification of 400×. Digital images of each microscopic field were captured, stored in a computer, and subsequently analyzed for stain density using free ImageJ software (US National Institutes of Health, Bethesda, Maryland, USA) and the methods described by Girish and Vijayalakshmi [15]. The immunostaining density of HIF-1α and VEGF in each experimental group was expressed as a proportion of the corresponding control sections.

### Assessment of the vascularization of the corpora lutea

Three additional alternate sections of each ovary were processed by conventional histological methods and stained with H–E to evaluate the corpus luteum area occupied by vasculature. Five fields of each section, selected at random, were observed by light microscopy at a magnification of 400×. The obtained images were analyzed using ImageJ software and the above methods [15]. The area occupied by the corpus luteum vasculature was expressed as a percentage of the total area of luteal tissue.

A summary of the experimental design is presented in Table 2.

**Table 2:** Experimental design

**Experimental day**	**Experimental procedure**
*0*	Transfer of low-altitude native sheep to a high altitude experimental station and formation of the experimental groups (HH: n=6; HHV: n=6; LH: n=6; LHV: n=6; LL: n=6; LLV: n=6)
*5*	Start treatment with vitamins C and E in groups HHV, LHV and LL
*5*	Surgery for installation of the arterial and venous catheters
*6*	Administration of the first dose of cloprostenol for estrus synchronization
*15*	Administration of the second dose of cloprostenol for estrus synchronization
*15*	Introduction of vasectomized males for detection of females in estrus
*17-18*	First estrus after synchronization treatment
*34-36*	Second estrus after synchronization treatment
*34-54*	Daily sampling of arterial blood for evaluation of oxygenation status and venous blood for the measurement of progesterone and biomarkers of oxidative stress (n=6 each group)
*34-54*	Daily ultrasound examinations for evaluation of preovulatory follicle size and corpus luteum growth (n=6 each group)
*51-54*	Third estrus after synchronization treatment
*56-59*	Laparoscopic removal of corpora lutea for assessment of growth factors and vascularization (n=6 each group)

In the representation of the experimental groups, the first letter of the acronym is the altitudinal origin and the second letter is the altitude where the animals stayed during the study (H for high altitude, L for low altitude). If the animal were supplemented with vitamins, the letter V was added. Therefore, there are three unsupplemented groups (HH, LH and LL) and three vitamin-supplemented groups (HHV, LHV and LLV).

### Statistical analysis

The data describing most of the experimental variables, except for the gene expression, were compared by analysis of variance using the general linear model procedure (GLM; SAS Institute Inc., Cary, NC, USA). Comparisons were made using two statistical models. The first model was used to test the effect of altitude, including the two following cross factors: the place of birth or origin of the animals and the place where the reproductive cycles were studied. The second model took into consideration the previous two factors in addition to the antioxidant vitamin supplementation. The interactions among these factors were also analyzed. When significant differences were found, Duncan’s test was used to determine the groups among which the differences were statistically significant. For variables expressed as percentages, the results were compared after normalization of the data by means of angular transformation. The gene expression results were analyzed using a non-parametric Kruskal-Wallis test and a Dunn`s multiple comparison test after normalization of the data. Additionally, correlation analyses to establish the association between the studied traits were performed. A probability of P≤ 0.05 was considered statistically significant. The results are expressed as the means ± SEM.

## Results

### Effects of altitude, altitudinal status and antioxidant treatment on oxygenation status and oxidative stress

The assessment of arterial blood gases and other variables related to oxygen transport are shown in Table 3. Significant differences were found for all of the variables among the groups with different origins and altitudinal statuses. Overall, the animals exposed to high altitude showed blood changes consistent with a state of hypoxemia (low arterial oxygen pressure, low saturation of hemoglobin by oxygen and high hematocrit and hemoglobin concentrations). In contrast, the antioxidant vitamin treatment had no significant effect on any of the parameters evaluated, as revealed by the finding that the improvement in the P_a_O_2_ and the saturation of Hb in the high-altitude naïve sheep did not reach statistical significance.

**Table 3:** Blood gases in cycling sheep at a high altitude: The effect of supplementation with vitamins C and E

**Group**	**P**_**a**_**O**_**2**_	**P**_**a**_**CO**_**2**_	**Hb**	**Sat Hb**	**Ht**	**pH**
	**(mm Hg)**	**(mm Hg)**	**(mg/dL)**	**(%)**	**(%)**	
*LL*	97.0±5.6^a^	39.4±1.5^a^	10.5±0.4^b^	97.6±3.1^a^	30.5±1.3^de^	7.46±0.04^c^
*LLV*	97.2±2.8^a^	39.9±2.1^a^	10.7±1.1^b^	95.8±6.1^a^	29.7±1.3^e^	7.45±0.03^bc^
*LH*	53.3±11.9^c^	38.9±8.5^a^	12.4±1.0^a^	68.9±21.0^c^	37.4±3.1^ab^	7.46±0.07^bc^
*LHV*	58.0±5.0^bc^	35.4±2.4^ab^	12.9±1.2^a^	75.4±5.0^bc^	38.8±3.6^a^	7.50±0.04^ab^
*HH*	60.3±4.2^b^	34.4±3.8^b^	11.7±1.2^ab^	84.2±5.5^b^	35.2±3.4^bc^	7.51±0.03^a^
*HHV*	60.1±4.2^b^	33.3±4.1^b^	11.0±1.0^b^	81.5±4.3^b^	33.2±3.0^cd^	7.52±0.04^a^

Different superscript letters indicate significant differences among groups (P<0.05, Duncan’s test). P_a_O_2_: arterial pressure of oxygen; P_a_CO_2_: arterial pressure of carbon dioxide; Hb: hemoglobin concentration: Sat Hb: saturation of hemoglobin by oxygen; Ht: hematocrit.

LL: low altitude native sheep maintained at a low altitude, without vitamin supplementation; LLV: low altitude native sheep maintained at a low altitude, supplemented with vitamins; LH: low altitude native sheep taken to a high altitude, without vitamin supplementation; LHV: low altitude native sheep taken to a high altitude, supplemented with vitamins; HH: high altitude native sheep maintained at a high altitude, without vitamin supplementation; HHV: high altitude native sheep maintained at a high altitude, supplemented with vitamins.

**Figure 1:**
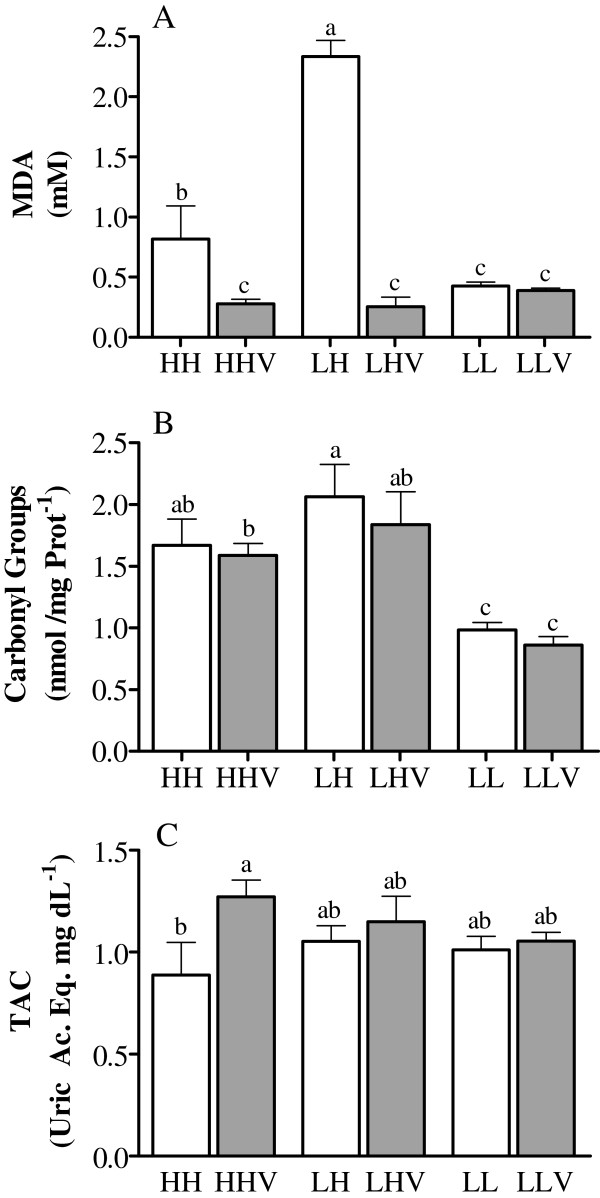
**Oxidative stress biomarkers in cycling sheep at a high altitude.** Malondialdehyde (panel **A**), carbonyl groups (panel **B**), and the total antioxidant capacity (panel **C**) were measured in cycling sheep at a high altitude that were either unsupplemented (HH: high-altitude natives; LH: low-altitude natives) or supplemented daily (HHV: high-altitude natives; LHV: low-altitude natives) with vitamins C and E. Groups LL and LLV correspond to the control cycling sheep at a low altitude without and with vitamin supplementation, respectively. The different letters above the columns indicate significant differences among the groups (P<0.05, Duncan`s test).

The oxidative damage biomarkers are shown in Figure 1. The plasma malondialdehyde concentrations were highest in sheep from the LH group (P<0.05), and they were higher in the HH group than in the LL group (P<0.05). Conversely, the protein carbonyl groups were similar in the HH and LH ewes and higher in both of these groups than in the LL group (P<0.05). Finally, no significant differences were found in the total antioxidant capacity between the three groups of sheep. The administration of antioxidants reduced the malondialdehyde concentrations in the HH and LH females to levels similar to those in the LL ewes, but this treatment only improved the total antioxidant capacity in HH females (P<0.05). The carbonyl levels were not affected in any group.

### Effects of altitude, altitudinal status and antioxidant treatment on the pattern of growth, vascularization and functionality of the corpora lutea

**Figure 2:**
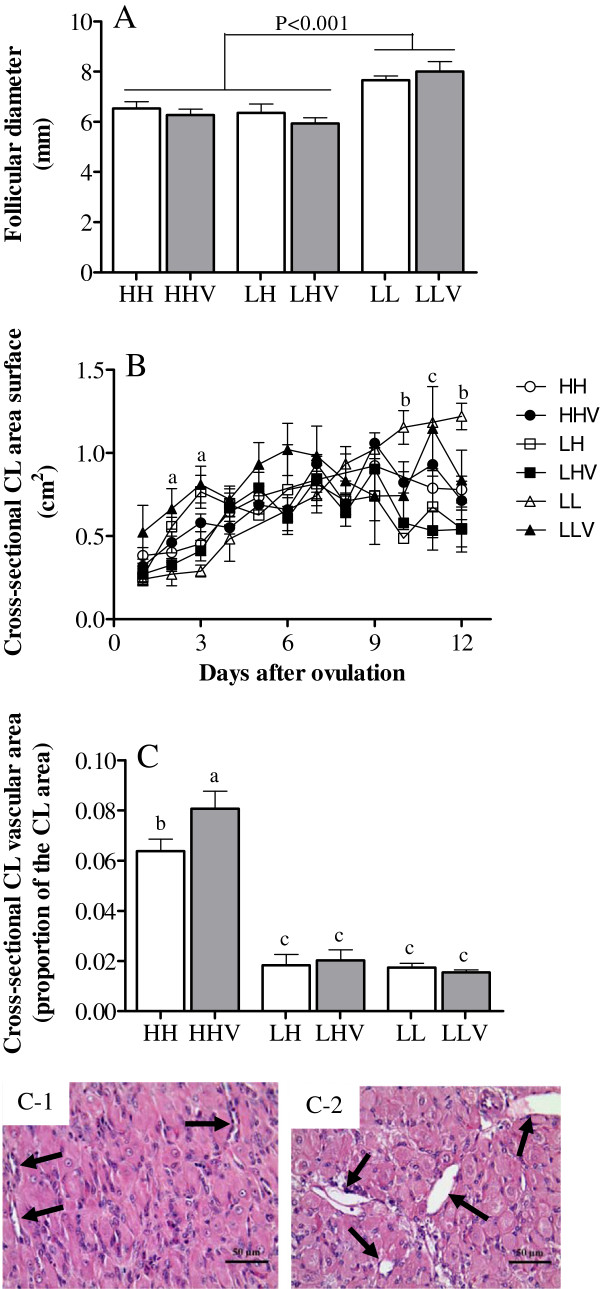
**Characteristics of the main ovarian structures in cycling sheep at a high altitude.** The maximum preovulatory follicular diameter (panel **A**), corpus luteum area dynamics (panel **B**) and corpus luteum vascular area (panel **C**) were determined in cycling sheep at a high altitude either unsupplemented (HH: high-altitude natives; LH: low-altitude natives) or supplemented daily (HHV: high-altitude natives; LHV: low-altitude natives) with vitamins C and E. Groups LL and LLV correspond to the control cycling sheep at a low altitude without and with vitamin supplementation, respectively. Sub-panels C-1 and C-2 show representative images of groups where minor and major cross-sectional CL vascular areas were obtained, respectively (arrows show blood vessels; magnification 400x). In panel B, the letters above the symbols indicate significant differences among groups at the same time interval (P<0.05) as follows: a, groups LLV and LH are higher than all of the other groups; b, group LL is higher than all of the other groups; c, groups LL and LLV are higher than all of the other groups. In panel C, the different letters above the columns indicate significant differences among groups (P<0.05, Duncan`s test).

The mean ovulation rate was 1.26±0.48, and there were no differences among the groups (P=0.08; Table 4 show the average of corpora lutea in each group). The mean size of the ovulatory follicles in the LL group was significantly larger (P<0.001) than in the HH and LH groups, and no effect of the antioxidant treatment was detected (Figure 2A). After ovulation, the overall size of the luteal tissue throughout the luteal phase was similar in all six groups (Figure 2B), except for the area of the luteal tissue in the LH and LLV groups. In those groups, the areas were significantly larger than in all of the other groups at days 2 and 3, whereas the LL group showed the largest area on days 10, 11 and 12 after ovulation (P<0.05). The LLV group was similar to the LL group on day 11.

**Table 4:** Number of corpora lutea in cycling sheep at a high altitude

**Experimental group**	**Number of corpora lutea**
*LL*	1.33 ± 0.52
*LLV*	1.17 ± 0.41
*LH*	1.33 ± 0.52
*LHV*	1.50 ± 0.55
*HH*	1.17 ± 0.41
*HHV*	1.33 ± 0.52

LL: low altitude native sheep maintained at a low altitude, without vitamin supplementation; LLV: low altitude native sheep maintained at a low altitude, supplemented with vitamins; LH: low altitude native sheep taken to a high altitude, without vitamin supplementation; LHV: low altitude native sheep taken to a high altitude, supplemented with vitamins; HH: high altitude native sheep maintained at a high altitude, without vitamin supplementation; HHV: high altitude native sheep maintained at a high altitude, supplemented with vitamins.

The corpus luteum area occupied by vasculature was significantly affected by the origin of the ewes, and the vascularized area was greater in the HH sheep than in the LL and LH sheep (P<0.001), without significant effects of altitudinal status (LL and LH sheep with similar CL area; P=0.845). In this case, the supply of antioxidant agents increased vascularization in the HHV group (P<0.05), but it had no effect in the other two groups (Figure 2C).

**Figure 3:**
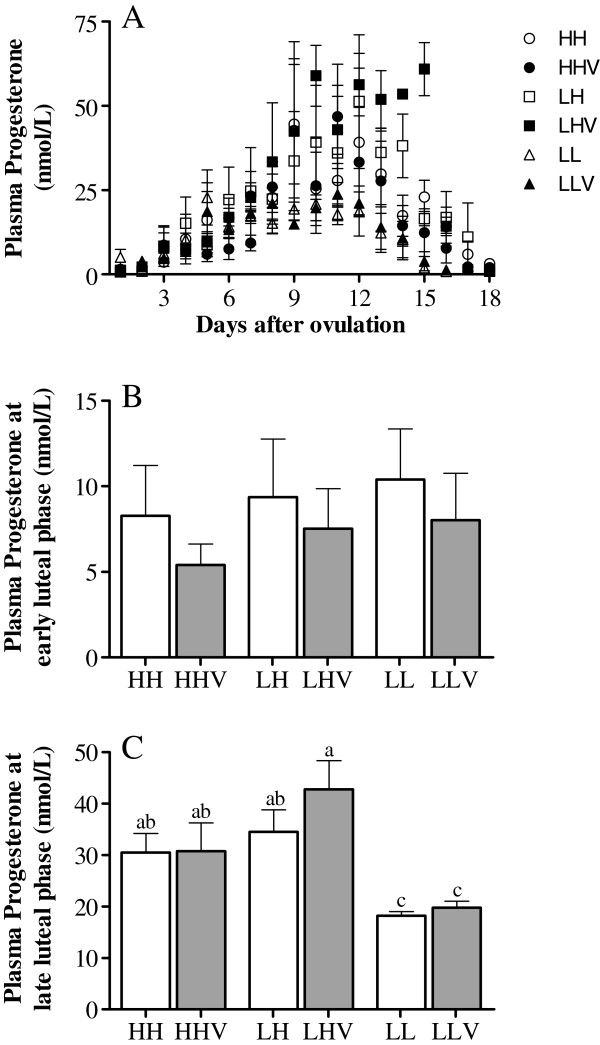
**Plasma progesterone concentrations in cycling sheep at a high altitude.** Progesterone profiles were determined during the entire non-conceptional reproductive cycle (panel **A**) in sheep at a high altitude that were either unsupplemented (HH: high-altitude natives; LH: low-altitude natives) or supplemented daily (HHV: high-altitude natives; LHV: low-altitude natives) with vitamins C and E. Groups LL and LLV correspond to the control cycling sheep at a low altitude without and with vitamin supplementation, respectively. Panels **B** and **C** represent the progesterone group averages during the early (days 1 to 6 after ovulation) or late (days 7 to 12 after ovulation) luteal phase of the same reproductive cycles. The different letters above columns indicate significant differences among groups (P<0.05, Duncan`s test).

The assessment of the pattern of plasma progesterone concentration during the entire non-conceptional reproductive cycle (Figure 3A) showed no significant differences between different altitude exposures and altitudinal statuses, and the plasma progesterone concentration was not affected by the antioxidant treatment. Similarly, we found no significant differences when comparing the mean daily values during the early luteal phase (days 1 to 6 after ovulation; Figure 3B). However, altitude significantly affected progesterone secretion during the late luteal phase (days 7 to 12 after ovulation; Figure 3C), and the plasma progesterone levels were higher in the HH and LH ewes than in the LL ewes (P<0.05). Conversely, again, the antioxidant treatment showed no effect.

### Effects of altitude, altitudinal status and antioxidant treatment on the corpus luteum IGF-I and IGF-II gene expression

**Figure 4:**
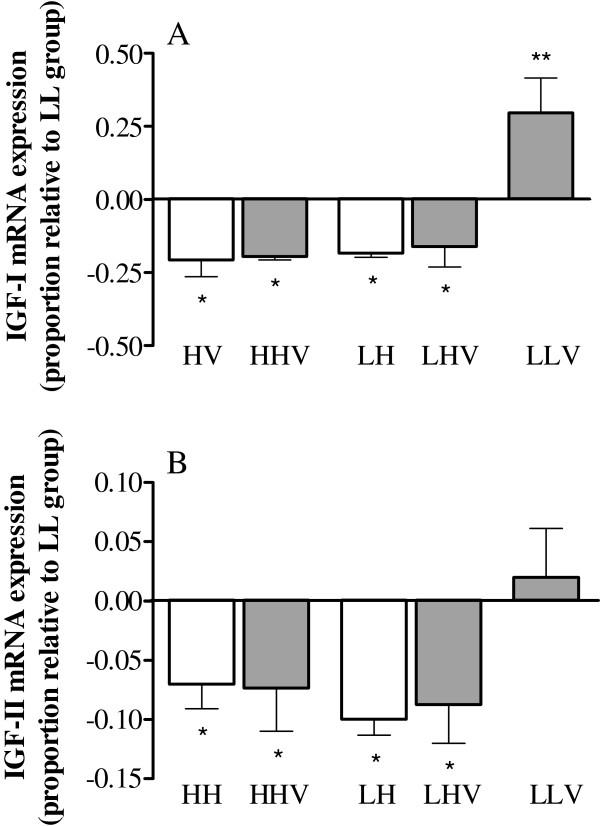
**Luteal expression of insulin-like growth factors in cycling sheep at a high altitude.** IGF-I (panel **A**) and IGF-II (panel **B**) mRNA expression (2^-ΔΔCt^) in luteal tissue obtained at day 5 after ovulation in non-conceptional reproductive cycles in sheep at a high altitude that were either unsupplemented (HH: high-altitude natives; LH: low-altitude natives) or supplemented daily (HHV: high-altitude natives; LHV: low-altitude natives) with vitamins C and E. The LLV group corresponds to control cycling sheep at a low altitude with vitamin supplementation. The values of mRNA expression in each group are expressed relative to the control group LL (low-altitude native ewes maintained at a low altitude). Asterisks indicate significant differences (*= P<0.01 and ** = P<0.001) when compared to the control LL group (Dunn`s multiple comparison test).

The exposure to high altitude affected luteal IGF-I and IGF-II mRNA expression (Figure 4). Thus, the expression of both growth factors was significantly decreased in the four groups kept at a high altitude (HH, HHV, LH and LHV) when compared to the LL and LLV groups kept at a low altitude (P=0.004). In contrast, vitamin supplementation only affected IGF-I expression (P=0.022), with the LLV group showing a higher level of IGF-I expression (Figure 4A). The altitudinal status showed no significant effect on either growth factor. Furthermore, significant correlations between the area of the corpus luteum and the luteal expression of IGF-I (r=0.614; P=0.05) and IGF-II (r=0.555; P=0.032) were found.

### Effects of altitude, altitudinal status and antioxidant treatment on HIF-1α and VEGF immunohistochemical expression in the corpus luteum

**Figure 5:**
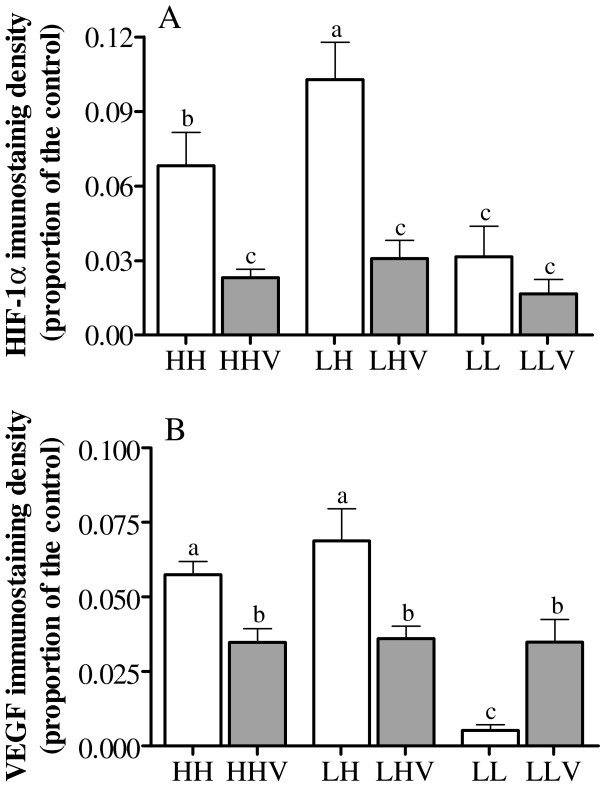
**Hypoxia inducible factor 1α and vascular endothelial growth factor luteal immunoreactivity in cycling sheep at a high altitude.** HIF-1α (panel **A**) and VEGF (panel **B**) immunostaining density were evaluated in corpora lutea at day 5 after ovulation in non-conceptional reproductive cycles in sheep at a high altitude either unsupplemented (HH: high-altitude natives; LH: low-altitude natives) or supplemented daily (HHV: high-altitude natives; LHV: low-altitude natives) with vitamins C and E. Groups LL and LLV correspond to the control cycling sheep at a low altitude without and with vitamin supplementation, respectively. The different letters above the columns indicate significant differences among the groups (P<0.05, Duncan`s test).

**Figure 6:**
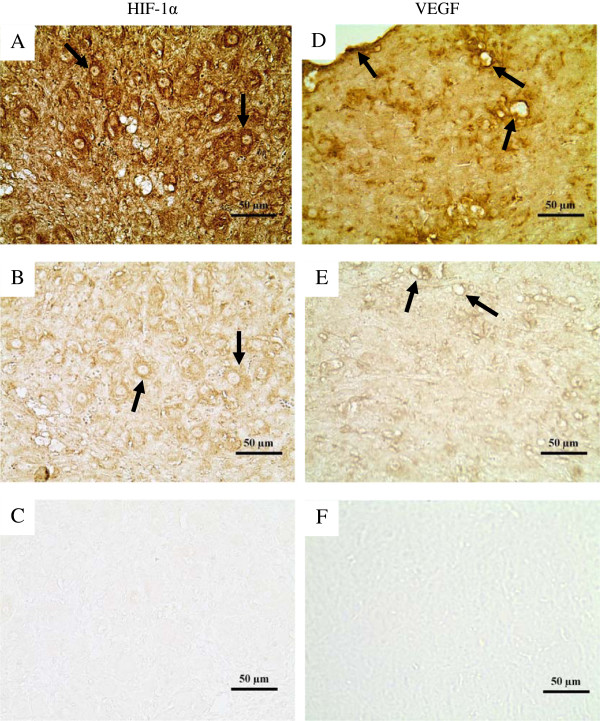
**Representative images of immunohistochemical expression of hypoxia inducible factor 1α and vascular endothelial growth factor in corpora lutea from cycling sheep at a high altitude.** Images **A** and **B** show sections of luteal tissue with high and low HIF-1α expression, respectively. The arrows show examples of luteal cells expressing the protein. Image **C** corresponds to a control section prepared in the absence of HIF-1α antibody. Images **D** and **E** show sections of luteal tissue with high and low VEGF expression, respectively. The arrows show blood vessels where VEGF staining was denser. Image **F** corresponds to a control tissue section prepared in the absence of VEGF antibody. Magnification 400×.

The immunohistochemical expression of HIF-1α and VEGF in the luteal tissue is shown in Figure 5, and representative images are shown in Figure 6. HIF-1α in the luteal tissue (Figure 5A) was significantly affected by the altitude and the altitudinal status; this measure was higher in HH and LH sheep than in LL sheep and was highest in the LH group (P<0.001). Vitamin supplementation decreased HIF-1α expression in both HH and LH sheep (P<0.001). However, no effect of vitamin supplementation was observed in the LLV group, which may be related to the statistically significant interaction between the altitudinal status and vitamin supplementation (P=0.014).

In the case of immunohistochemical expression of VEGF in the corpora lutea (Figure 5B), a significant effect of altitude was observed, with HH and LH sheep showing a higher level of expression than LL sheep (P<0.001). In addition, vitamin supplementation was associated with a significant decrease in VEGF expression in the HHV and LHV sheep (P<0.01). The statistically significant interaction between the altitudinal status and vitamin supplementation (P<0.001) may explain the opposite effect found in the LLV group.

## Discussion

The results of the present study clearly indicate changes in the growth and functionality of luteal tissue induced by both long- and short-term exposure to high altitude.

The corpora lutea of sheep that were native and naïve to a high altitude were, overall, smaller in size than the corpora lutea of ewes at a low altitude. The occurrence of growth deficiencies in the corpora lutea has been classically linked to ovarian causes (inadequate development and maturation of preovulatory follicles [16]) and/or systemic causes (inadequate LH secretion, which is necessary for the final maturation of the preovulatory follicles and, subsequently, adequate development of the corpora lutea [17,18]). The data obtained in the current experiment indicate the existence of alterations in preovulatory follicle development as a result of either short- or long-term exposure to high-altitude environments, which supports previous findings in humans [19]. However, the evaluation of possible alterations in the pituitary secretion of LH requires further study.

The altered growth of the corpora lutea in ewes exposed to a high altitude was confirmed by analysis of both the expression of key factors involved in corpora lutea growth and vascularization and of the proper vascularization of the structure. First, the exposure to hypoxia negatively affected the expression of both IGF-I and IGF-II, which are essential for the early development and function of the corpora lutea [20-22]. In fact, the levels of IGFs were correlated with the area of luteal tissue. Moreover, a larger size of the corpus luteum in the late luteal phase was observed in the LL group. Second, there were differences in the vascularization of the corpora lutea and the expression of growth factors driving this vascularization; in this case, these parameters were increased in sheep kept at a high altitude. The early developmental stages of a corpus luteum are characterized by rapid changes in the cells of the ovulatory follicle after ovulation, involving proliferation, differentiation and transformation [23], which require a well-developed vascular system to fulfill the increased metabolic demands of the cells and, at the same time, distribute the secreted hormones throughout the body [24]. A close relationship among vascularization, blood flow and progesterone production and secretion has been reported in the bovine corpus luteum [25]. In the current study, this relationship between luteal vascularization and progesterone production was observed in the sheep studied in their place of origin (groups HH and LL). The production of VEGF, one of the main angiogenic factors, is highly stimulated by hypoxia [26]. Elevated VEGF expression and production in human luteal tissues cultured under hypoxic conditions have been demonstrated [27]. Similar mechanisms would explain the increases in luteal VEGF expression and vascularization in the sheep of the present study. However, sheep that were naïve to a high altitude and exposed to a high altitude for a short time period (group LH) showed the highest progesterone production in the absence of corresponding luteal vascularization, despite the increased amounts of VEGF. Thus, the classical concept that the growth and the secretory functions of the developing corpus luteum require intense angiogenesis and stabilization of blood vessels for adequate regulation of luteal function [28] does not appear to be the rule for sheep acutely exposed to high-altitude hypoxia. The apparently exceptional response of this group to altitude exposure (increased expression of HIF-1α and VEGF, decreased expression of IGF-I and IGF-II, increased levels of progesterone and null angiogenic response) may represent an earlier and most likely incomplete response to hypoxia due to the short lifespan of the corpus luteum, which may limit the response to hypoxia. Consistent with this hypothesis, it has been observed that the placenta (a steroidogenic tissue with a longer lifespan than the corpus luteum) secretes more progesterone in pregnant sheep naïve to high altitude than in sheep native to high altitude conditions [6], but the placental vascularization increases similarly in naïve and native sheep in response to the augmented VEGF expression [29].

Thus, the most significant and striking finding in the current experiment is the increased level of progesterone found during the late luteal phase in the ewes exposed to a high altitude, which was double the value obtained in sheep located at a low altitude. This effect was observed in both vitamin-supplemented and non-supplemented sheep, despite a smaller size of the corpus luteum. Plasma progesterone concentrations in the females living at a low altitude remained at values similar to those described elsewhere [30]. No information about the effect of altitude exposure on progesterone levels during animal reproductive cycles was found in the literature. However, our results are consistent with those reported for women living at a high altitude. Indigenous non-pregnant women residing at a high altitude had greater progesterone levels during the luteal phase of the menstrual cycle in comparison with women living at sea level [31]. Additionally, women exposed to acute hypobaric hypoxia showed higher plasma progesterone concentrations during the luteal phase than controls under normoxia [32].

Further studies are necessary to determine the possible causes for such an increase in progesterone levels. A possible cause is an increased vascularization of the corpora lutea and, accordingly, an increased secretion of progesterone to the peripheral blood flow [33]; however, as previously argued, this explanation does not entirely fit with the observations in high-altitude naïve animals. Most likely, the differences result from variations in progesterone metabolism rather than in secretion. Plasma progesterone levels are known to be more affected by metabolic clearance than by the level of secretion from the luteal tissue [34]. As such, the studies performed in women exposed to hypoxia showed decreased steroid clearance [35]. Another possible explanation is a masking effect of progesterone, which is known to stimulate respiration and to protect against breathing disorders [36]. Thus, it is possible that females secrete higher levels of progesterone to better adapt to hypoxia; significant increases in plasma progesterone concentrations at high altitudes have even been described in men [37].

The existence of augmented plasma progesterone concentrations during the late luteal phase may compromise female fertility by affecting the final development and maturation of the preovulatory follicle of the subsequent cycle. Furthermore, alterations in follicular development diminish a follicle’s ability to ovulate an oocyte that can be fertilized and develop into a viable embryo [38]. Progesterone exerts an inhibitory action on gonadotropin-releasing hormone release from the hypothalamus [39], resulting in inadequate stimulation of gonadotrophs for LH synthesis. The hormone LH is pivotal for final maturation of preovulatory follicles [18]; progesterone levels that are too high may interfere with this process. In sheep, as in other species, it is well known that large follicles have lower functionality in the mid-luteal phase [30] when progesterone concentrations are at the maximum level. Ovulation in defective follicles results in lower fertility [40], which can contribute to the lower fertility observed at a high altitude.

Antioxidant vitamin supplementation, such as that in the present experiment, has shown significant and beneficial effects on sheep gestation at high altitudes, specifically improving pregnancy outcomes and newborn lamb parameters [2,7]. These effects are associated with an attenuation of the hypoxic response at the placental level, both in size and vascularization, as well as in blood oxygenation and oxidative stress biomarkers [7]. In addition, the daily supplementation of antioxidant vitamins during pregnancy significantly increased plasma progesterone concentrations in sheep living at a high altitude [6]. In the present experiment, this same effect appears to be present in high-altitude naïve sheep, though statistical significance was not reached. Long-term treatment with antioxidant vitamins is most likely necessary to significantly increase the magnitude of the augmentation of steroidogenesis. This requirement might explain the different responses to vitamin supplementation between a steroidogenic tissue with a short lifespan (corpus luteum) and a tissue with a long lifespan (placenta) in sheep exposed to a high altitude. In the current experiment, vitamin supplementation showed limited effects, which were mainly attributed to diminished levels of oxidative stress biomarkers and attenuated corpus luteum HIF-1α and VEGF responses to hypoxia, without a correlation with corpus luteum vascularization. These discordant results allow us to propose that, in contrast to the placenta, the sheep corpus luteum may be a structure that is partially protected against oxidative stress. Alternatively, the effects of hypoxia may substantially exceed those of oxidative stress, thereby masking the antioxidant effects. However, these alternative hypotheses require detailed study.

## Conclusions

Exposure of sheep to high-altitude hypobaric hypoxia for short or long time periods affects corpus luteum development and function, which may help explain the decreased sheep fertility at a high altitude. The presence of a state of oxidative stress associated with hypoxia, together with the absence of a significant effect of antioxidant vitamins on most of the anatomical and functional corpus luteum traits, suggests that the effects of high altitude on this ovarian structure are mainly mediated by hypoxia.

## Competing interests

The authors declare that they have no competing interests.

## Authors’ contributions

VHP, BU, SA and AGB were responsible for the study design, data collection, analysis of the results and writing of the manuscript. LP participated in this study as a postgraduate student. She was responsible for the supervision of the animals during the experiment, the sampling, the development of radioimmunoassay techniques and the analysis of the results. LTR and AAM were responsible the laboratory techniques and critical revision of the manuscript. GC and MDlR collaborated in the analysis and interpretation of the results and the correction and critical review of the manuscript. All authors read and approved the final version of the manuscript.
